# PP53 Potential Cost-Effectiveness And Innovation Headroom Of A More Accurate Companion Diagnostic For PD-L1 In Non-Small-Cell Lung Cancer

**DOI:** 10.1017/S0266462324002241

**Published:** 2025-01-07

**Authors:** Toluwase Akinsoji, Nick Dragojlovic, Cécile Darviot, Michel Meunier, Larry D. Lynd

## Abstract

**Introduction:**

While targeted therapies have substantially improved survival rates for non-small-cell lung cancer (NSCLC), it remains the leading cause of cancer mortality in the US. Companion diagnostics (CDx) measuring programmed death-ligand 1 (PD-L1) expression help inform NSCLC treatment but have limited accuracy. We assessed the potential value of a more accurate PD-L1 CDx for multiple stakeholders.

**Methods:**

We developed decision tree models to assess the potential cost-effectiveness of a hypothetical new CDx for atezolizumab, a PD-L1 inhibitor used as a first-line therapy for metastatic NSCLC and as an adjuvant therapy for stage II–IIIa NSCLC patients. The sensitivity and specificity of current PD-L1 assays as well as cost and health payoffs were estimated based on data extracted from the scientific literature. We calculated incremental cost-effectiveness ratios (ICERs) for both indications, conducted headroom and threshold analyses, and used model outputs to estimate the size of the US serviceable addressable market (SAM) for a new PD-L1 CDx in the adjuvant indication.

**Results:**

Approximately five percent of metastatic and seven percent of stage II–IIIa NSCLC patients currently tested for PD-L1 expression receive false negative results. An equivalently priced 100 percent accurate PD-L1 CDx would add an average of 0.04 quality-adjusted life years (QALYs) and cost USD6,069 more per metastatic NSCLC patient (ICER: USD144,512/QALY gained; 95% confidence interval [CI]: USD74,178, USD206,937). It would add 0.08 QALYs and cost USD3,682 more for stage II–IIIa NSCLC patients (ICER: USD49,031/QALY gained; 95% CI: USD47,104, USD50,064). The maximum value-based price in stage II–IIIa NSCLC (assuming a USD100,000/QALY willingness-to-pay threshold) would be ˜USD4,000/patient. At a USD500 price per unit, the SAM would be ˜USD6.5M/year.

**Conclusions:**

Because existing PD-L1 assays for NSCLC are optimized for specificity, the cost-effectiveness of a more accurate CDx reflects that of the immunotherapy drug it is paired with. Stage II–IIIa NSCLC has much greater innovation headroom for a new PD-L1 CDx than metastatic NSCLC. Early cost-effectiveness modeling can identify CDx use cases with higher value headroom and help inform market sizing.

